# Rotigotine Patch is Effective for Levodopa (L-Dopa)-Induced Dyspnea: A Case Report

**DOI:** 10.7759/cureus.85258

**Published:** 2025-06-02

**Authors:** Koji Hayashi, Yuka Nakaya, Koichi Kimura, Mamiko Sato, Hiromi Hayashi, Kouji Hayashi, Yasutaka Kobayashi

**Affiliations:** 1 Department of Rehabilitation Medicine, Fukui General Hospital, Fukui, JPN; 2 Graduate School of Health Science, Fukui Health Science University, Fukui, JPN

**Keywords:** dyspnea, oral levodopa, oral levodopa therapy, parkinson's disease, respiratory dysfunction

## Abstract

Parkinson's disease (PD) is a progressive neurodegenerative disorder with increasing prevalence. While commonly recognized motor symptoms include mobility limitations and balance problems, less attention has been given to respiratory complications related to levodopa (L-dopa) treatment, particularly dyspnea. This report describes an 88-year-old woman with a long-standing history of PD who presented with fever, dyspnea, and dysphagia after being treated with L-dopa. Upon admission, her neurological examination revealed significant motor impairments, and chest imaging indicated right diaphragmatic elevation and passive atelectasis. Despite adjustments to her treatment regimen, including switching from intravenous to nasogastric L-dopa administration, she experienced severe dyskinesias and respiratory distress. Given her history of L-dopa-induced respiratory issues, we transitioned her treatment to a transdermal rotigotine patch. Remarkably, the switch resulted in complete resolution of her respiratory symptoms and dyskinesia, alongside improvements in her overall motor functions and activities of daily living. This case underscores the potential effectiveness of rotigotine as a treatment strategy for L-dopa-induced dyspnea, suggesting it may maintain stable blood concentrations and mitigate dyskinesias. Further investigation is warranted to explore the underlying mechanisms of L-dopa-induced respiratory depression and validate the therapeutic role of rotigotine in these cases.

## Introduction

Parkinson's disease (PD) is a progressive neurodegenerative disorder affecting about 1% of individuals over 60 years old [1]. The prevalence of PD has significantly increased, rising from 2.5 million in 1990 to approximately six million in 2016, making it the fastest-growing neurological disease globally [2,3]. A recent analysis suggests that the actual prevalence of PD may be underestimated by 50%, with a new diagnosis occurring every six minutes [4]. A diagnosis of PD often leads to functional disability and impaired quality of life [5]. While common symptoms include mobility limitations, difficulty with transfers, progressive balance problems, and significant walking challenges [6], dyspnea related to levodopa (L-dopa) treatment is less recognized.

Rotigotine is a non-ergoline dopamine agonist developed for the once-daily treatment of PD [7]. It is administered using a transdermal delivery system (patch) that provides continuous drug delivery over 24 hours [7]. Although there is clear evidence that this drug improves motor symptoms of PD [7], there have been no reports of improvement in respiratory symptoms, particularly L-dopa-induced dyspnea.

In this report, we describe a rare case of L-dopa-induced dyspnea and the successful treatment of this respiratory dysfunction using a transdermal rotigotine patch.

## Case presentation

An 88-year-old woman with a history of PD, diagnosed at age 69 in another hospital, presented to our hospital with fever, dyspnea, and dysphagia. She had been treated with oral medications, including anti-Parkinsonian agents, on an outpatient basis for 10 years following her diagnosis, but was admitted to a nursing home at the age of 79. Prior to admission to the nursing home, the patient was taking a combination of L-dopa/carbidopa 400/43.2 mg and 0.5 mg of pramipexole for PD. Following her admission to the nursing home, she was no longer under the care of a neurologist. Although detailed information on her medication history during this period was not available, she was receiving treatment with 300 mg of L-dopa/carbidopa immediately prior to this episode and was classified as Hoehn and Yahr (H-Y) stage V.

**Figure 1:**
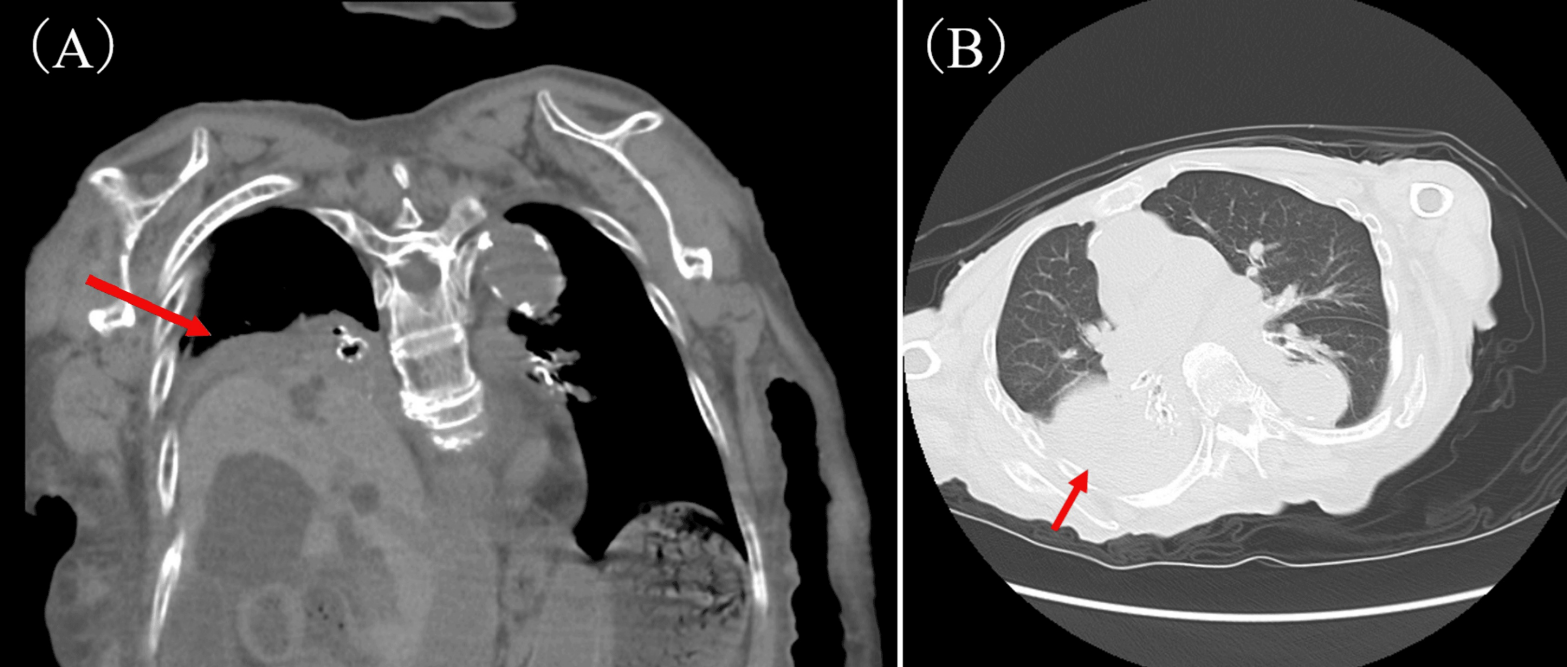
Thoracic CT scan Chest CT scan (A: coronal section; B: axial section) showing right diaphragmatic elevation, atelectasis, and a small pleural effusion (arrows).

On admission, vital signs were documented as follows: temperature of 37.4°C, blood pressure of 95/55 mmHg, and heart rate of 82 beats per minute. The neurological examination revealed mutism, a strongly retroflexed neck, rigidospasticity with contractures in the neck, limbs, and trunk, and hyperactive reflexes in the jaw and limbs. Blood test results showed slightly elevated C-reactive protein (CRP) as well as significantly decreased hemoglobin, total protein, albumin, alanine aminotransferase, γ-glutamyltransferase, sodium, and potassium (Table 1). Chest CT revealed the right diaphragmatic elevation and passive atelectasis (Figure 1). Although the patient was diagnosed with dysregulation of body temperature, likely due to autonomic nervous system dysfunction associated with end-stage PD, antibiotic treatment was initiated because of a slight increase in CRP, and the possibility of pneumonia in the atelectatic areas could not be ruled out.

**Table 1:** Results of blood tests upon admission

Inspection items	Result	Reference range
White blood cell count	8.0×10^3^ /μl	(3.3–8.6×10^3^)
Red blood cell count	307×10⁴ /μl	(386–492×10⁴)
Hemoglobin	10.3 g/dl	(11.6–14.8)
Blood platelet	22.6×10⁴ /μl	(15.8–34.8)
Total protein	5.8 g/dl	(6.6–8.1)
Albumin	3.1 g/dl	(4.1–5.1)
Glucose	108 mg/dl	(73–109)
Blood urea nitrogen	18.8 mg/dl	(8.0–20.0)
Creatinine	0.65 mg/dl	(0.46–0.79)
Total bilirubin	0.4 mg/dl	(0.4–1.2)
Aspartate aminotransferase	16 U/l	(13–30)
Alanine aminotransferase	4 U/l	(7–30)
Alkaline phosphatase	64 U/l	(38–113)
Lactate dehydrogenase	150 U/l	(124–222)
γ-glutamyltransferase	7 U/l	(13–64)
Creatine phosphokinase	45 U/l	(41–153)
Choline esterase	288 U/l	(240–421)
Amylase	83 U/l	(44–132)
Sodium	137 mmol/l	(138–145)
Potassium	3.5 mmol/l	(3.6–4.8)
Chlorine	102 mmol/l	(101–108)
C-reactive protein	0.65 mg/dl	(0.00–0.14)

Swallowing assessments and rehabilitation treatment were planned. An initial swallowing screening by a speech therapist upon admission indicated difficulty with oral medication intake, leading to the prescription of intravenous L-dopa (100 mg once, twice daily). About 30 minutes following each L-dopa infusion, significant dyskinesia developed in the neck and limbs, accompanied by wheezing and a decrease in peripheral oxygen saturation (SpO_2_) (below 90%) for one hour, requiring oxygen treatment. Suspecting side effects specific to the intravenous route, the medication was changed to L-dopa administration via a nasogastric tube (L-dopa/carbidopa 300 mg). However, similar dyskinesia, wheezing, and decreased SpO_2_ were observed approximately 60 minutes after oral administration, lasting for about 1-2 hours. Blood gas analysis under oxygen therapy revealed respiratory acidosis (Table 2).

**Table 2:** Blood gas analysis under oxygen treatment after admission pCO_2_: Partial pressure of carbon dioxide; pO_2_: Partial pressure of oxygen; HCO_3_^-^: Bicarbonate

Inspection items	Result	Reference range
pH	7.37	(7.38–7.46)
pCO_2_	54.6 Torr	(32.0–46.0)
pO_2_	80.0 Torr	(74.0–108.0)
HCO_3_^-^	31.7 mmol/l	(21.0–29.0)
Base excess	5.9 mmol/l	(-2.0–2.0)

Suspecting L-dopa-induced respiratory dysfunction, L-dopa was switched to a rotigotine patch (18 mg/day). These symptoms completely resolved with the use of the rotigotine patch. A swallowing assessment while using the rotigotine patch revealed no difficulties with consuming items such as jelly. Rotigotine was considered to have contributed, at least in part, to the improvement in her motor symptoms. She no longer had fevers, and it was determined that, following this medication adjustment, she had returned to her pre-hospital level of activities of daily living (ADLs).

## Discussion

This case report presents the first instance of L-dopa-induced respiratory dysfunction in a patient with end-stage PD (H-Y stage V) being successfully controlled with rotigotine patches. The patient had experienced dyskinesia and respiratory failure shortly after L-dopa administration. Rotigotine was used while L-dopa was discontinued, demonstrating a degree of efficacy in managing PD-related motor symptoms, and effectively controlled the respiratory dysfunction. Regarding the rotigotine dosage, previous studies indicate that 30 times the amount of rotigotine is equivalent to L-dopa in terms of potency [8]. Considering the patient's overall decline in ADLs and undertreatment due to insufficient L-dopa dosage, rotigotine 18 mg/day was selected to increase the L-dopa equivalent.

Respiratory symptoms, although not a frequent complication of PD, have been reported to occur with disease progression, often attributed to factors such as upper airway obstruction, restrictive respiratory dysfunction, abnormal central control of ventilation, and medication-related dysfunction [9,10]. While the exact mechanism underlying respiratory symptoms in PD remains unclear, they are often associated with disease progression or medication wearing off [10]. Although symptom relief is often observed with titration of L-dopa doses [11,12], L-dopa-induced respiratory dysfunction is not well recognized. Nonetheless, there are some reports of L-dopa-induced respiratory dysfunction.

De Keyser and Vincken reported a case of a 64-year-old woman with a two-year history of PD who experienced severe bradykinesia and rigidity but no prior respiratory symptoms [13]. Initial treatment with anticholinergics was ineffective. After starting L-dopa/benserazide, she developed significant shortness of breath and chest discomfort about two hours after each dose, with irregular breathing persisting for several hours. Adding the dopamine antagonist tiapride normalized her breathing and alleviated dyspnea without worsening Parkinsonian symptoms. Symptoms reappeared upon stopping tiapride and resolved when it was restarted. The authors suggested that the respiratory disturbance was linked to dopamine receptor supersensitivity, effectively managed by tiapride.

Ko et al. reported a case of a 78-year-old male with a 10-year history of PD and chronic obstructive pulmonary disease (COPD) [10]. He exhibited shortness of breath, bradykinesia, rigidity, and dyskinesia while receiving 900 mg/day of L-dopa. Notably, his shortness of breath did not improve with COPD therapy and worsened around his L-dopa dosing schedule, despite normal oxygen levels and no carbon dioxide retention. An L-dopa challenge test confirmed that respiratory symptoms, including significant dyspnea, worsened with increased L-dopa dosage. After reducing the L-dopa dose and adding a catechol-O-methyltransferase (COMT) inhibitor, his respiratory symptoms improved without worsening motor function. The authors discussed the etiology of L-dopa-induced dyspnea, suggesting that L-dopa sensitivity may significantly affect respiratory function.

van de Wetering-van Dongen et al. reported three cases of biphasic (subtherapeutic) L-dopa-induced respiratory dysfunction [14]. All three patients with PD experienced shortness of breath and other respiratory symptoms, specifically timed around their L-dopa medication intake, specifically before the "on" state and again shortly before needing their next dose. The authors analyzed these cases and suggested that respiratory dysfunction might be developed by subtherapeutic concentrations of L-dopa. L-dopa-induced respiratory dysfunction improved with increased dosage and frequency of administration, as well as the introduction of continuous 16-hour L-dopa subcutaneous infusion therapy.

Yan et al. reported a case of a 75-year-old female with a 10-year history of PD who experienced significant dyspnea following L-dopa administration [15]. The patient had been treated with escalating doses of L-dopa/benserazide, and her respiratory distress was linked to motor complications characterized by choreiform movements. The dyspnea typically occurred around an hour after taking L-dopa, coinciding with peak drug effects. The authors attributed the dyspnea to dyskinesia, a phenomenon they termed respiratory dyskinesia. Adjusting or stopping anti-Parkinsonian medications, consistent with the management of dyskinesia, resulted in improvement of the dyspnea.

In summary, based on previous reports, the etiologies of L-dopa-induced dyspnea may be attributed to the following mechanisms: dopamine receptor hypersensitivity, subtherapeutic concentrations of L-dopa, and dyskinesia. The underlying mechanism of dyskinesia involves an alteration in dopamine receptor sensitivity; therefore, although the reports describe these mechanisms differently, they may essentially represent the same underlying phenomenon. In addition, the medications used in these case reports included tiapride, COMT inhibitors, increased doses or frequency of L-dopa, and continuous subcutaneous L-dopa therapy. Aside from tiapride, which may suppress hypersensitivity of dopamine receptors, these treatments may have been strategies to stabilize blood L-dopa concentrations, focusing on continuous dopaminergic stimulation (CDS). Moreover, the discontinuation of anti-Parkinsonian medication was also considered as an option.

In our case, although pleural effusion and atelectasis were noted in the right lung, the patient's respiratory problems coincided with the timing of drug administration rather than being consistent throughout. Additionally, pneumonia or other respiratory issues were not identified. Despite a long history of PD, the patient was managed with relatively low doses of L-dopa, potentially below the therapeutic threshold. Additionally, she developed dyspnea as well as dyskinesia immediately after L-dopa treatment. This suggests two possible mechanisms for L-dopa-induced dyspnea in our case: dyskinesia and subtherapeutic concentrations of L-dopa. However, it remains unclear whether the dyspnea was primarily due to dyskinesia or undertreatment leading to biphasic respiratory dysfunction. While the temporal proximity between the administration of L-dopa and the onset of respiratory disturbance accompanied by dyskinesia suggests that the respiratory disturbance associated with dyskinesia is more likely, the increase in the amount of rotigotine in this case, functioning as a dopamine equivalent, means that it cannot be ruled out that the respiratory disturbance may have improved due to altered therapeutic sensitivity to L-dopa.

Nevertheless, as a therapeutic management strategy, the use of rotigotine patches would have been appropriate regardless of the mechanism of this symptom. Rotigotine is absorbed through the skin via the patch, so that its blood concentration remains constant, allowing CDS to be achieved [16]. In addition, Giladi et al. found that dyskinesia was infrequent during up to six years of transdermal rotigotine therapy in patients with PD, with most cases occurring in those also taking L-dopa [17]. Furthermore, only 5% of patients taking rotigotine alone reported dyskinesia, which was generally described as "not disabling" or "mildly disabling" [17]. On the other hand, since quetiapine is a dopamine receptor blocker, there is concern that it may worsen motor symptoms in PD. Therefore, the transdermal rotigotine patch may be a viable option for treating L-dopa-induced dyspnea.

There are two limitations in this report. First, we were unable to obtain detailed medical records regarding the PD treatments. Therefore, we could not describe the chronological order of the progression of the disease in relation to drug compliance, medication response, and the emergence of dyskinesia. Second, respiratory function tests, including spirometry, could not be performed because the patient was at H-Y stage V of PD. Therefore, we were unable to analyze the details of the respiratory failure immediately following L-dopa administration. If this information had been available, a more compelling presentation could have been made.

## Conclusions

Based on the literature, L-dopa-induced dyspnea may be caused by the following mechanisms: dopamine receptor hypersensitivity, subtherapeutic concentrations of L-dopa, and dyskinesia. Possible treatment options include approaches that focus on CDS to reduce fluctuations in blood concentrations and strategies aimed at suppressing dyskinesias. Rotigotine is an anti-Parkinsonian medication that is absorbed transdermally and has evidence supporting its ability to maintain constant blood concentrations. Additionally, rotigotine is associated with a reduced incidence of dyskinesias. We believe that rotigotine is a promising treatment option for L-dopa-induced respiratory depression. Furthermore, this medication may be especially useful in patients where L-dopa pharmacokinetics are difficult to manage or oral administration is problematic. Further research is needed to better understand the mechanisms of L-dopa-induced respiratory depression and the effectiveness of rotigotine in its treatment.
